# DO NOT DRAG THE CHILDREN DOWN: MUTUAL SUPPORT AMONG COMORBID COUPLES WITH STROKE SURVIVORS IN HOSPITAL

**DOI:** 10.1093/geroni/igae098.1454

**Published:** 2024-12-31

**Authors:** Yongxia Mei, Zhenxiang Zhang, Qingqing Zhang, Zhiwei Liu, Baoyu Fan, Yating Liu, Sixun Zhang

**Affiliations:** Zhengzhou University, Zhengzhou, Henan, China (People’s Republic); Zhengzhou University, Zhengzhou, Henan, China (People’s Republic); Zhengzhou University, Zhengzhou, Henan, China (People’s Republic); Zhengzhou University, Zhengzhou, Hunan, China (People’s Republic); Zhengzhou University, Zhengzhou, Henan, China (People’s Republic); Zhengzhou University, Zhengzhou, Henan, China (People’s Republic); Zhengzhou University, Zhengzhou, Henan, China (People’s Republic)

## Abstract

The phenomenon of mutual support among co-morbid couples of recurrent older stroke survivors during hospitalization is gaining attention. Despite their crucial role, little is understood about how they support each other. The study aimed to explore the experiences of co-morbid couples of older stroke survivors with recurrent stroke supporting each other during hospitalization. Conducted as a descriptive phenomenology study, data collection occurred in neurology departments of five tertiary-level hospitals from July to September 2023. Twenty-one couples aged between 61 and 72 were purposively sampled. Interview recordings were transcribed and analyzed by two independent coders using Colaizzi’s framework in NVivo 12, revealing 9 subthemes across 3 macrothemes. “Do not drag the children down” is a strong thread, which is linked to three macrothemes: (1) maintaining the couple’s relationship through mutual support, (2) mutual support to avoid burdening the children, and (3) providing support while struggling between ideals and reality. Healthcare professionals should give more consideration to co-morbid couples of older stroke survivors with recurrent stroke who adhere to the idea of not burdening their children when supporting each other, and should take into account the cultural characteristics of the conflicts and differences that may arise between couples of stroke survivors in the process of mutual support. It is crucial to provide them with individualized and tailored support and interventions that can help these couples achieve a more optimal balance in their mutual support.

